# Irregular Antenatal Care Attendance among Pregnant Women during COVID-19 Pandemic in a Tertiary Care Centre: A Descriptive Cross-sectional Study

**DOI:** 10.31729/jnma.7472

**Published:** 2022-09-30

**Authors:** Meenu Maharjan, Kamana Sen, Bibechan Thapa, Sushmita Shrestha, Aradhana Jayaswal, Alina Poudel, Prasnna Basnet, Sunita Rana, Sneha Chaudhary, Pratistha Shrestha, Kritick Bhandari

**Affiliations:** 1 Department of Gynaecology and Obstetrics, KIST Medical College Teaching Hospital, Imadol, Lalitpur, Nepal; 2 KIST Medical College Teaching Hospital, Imadol, Lalitpur, Nepal; 3 Department of Emergency Medicine, Kirtipur Hospital, Kirtipur, Kathmandu, Nepal

**Keywords:** *antenatal care*, *COVID-19*, *pregnant women*

## Abstract

**Introduction:**

The COVID-19 pandemic has made access to antenatal care services difficult, which could lead to serious implications for the health of mothers and fetus. There is limited study regarding its impact on pregnant women. This study aimed to find out the prevalence of irregular antenatal care attendance among pregnant women during the COVID-19 pandemic in a tertiary care centre.

**Methods:**

A descriptive cross-sectional study was carried out among pregnant women attending antenatal care visits at the Department of Gynaecology and Obstetrics in a tertiary care centre from 23 July 2021 to 5 September 2021. Ethical approval was granted by the Institutional Review Committee (Reference number: 077/078/67). Convenience sampling was done. Point estimate and 95% Confidence Interval were calculated.

**Results:**

Among 196 pregnant women, 49 (25%) (18.96-31.06, 95% Confidence Interval) had irregular antenatal care attendance during the COVID-19 pandemic.

**Conclusions:**

The prevalence of irregular antenatal care attendance during the COVID-19 pandemic was lower than other studies done in similar settings. Antenatal care is crucial to prevent maternal, fetal morbidity and mortality, hence uninterrupted antenatal care services should be provided even during crisis situation like COVID-19 pandemics.

## INTRODUCTION

Disruptions in maternal health services is a major concern since the beginning of COVID-19 pandemic.^1^ It has hindered maintenance of uninterrupted maternal and neonatal health services. Due to travel restrictions, lockdowns, compromised health facilities and fear of contracting the infection, pregnant women are unable to attend their antenatal care (ANC).^2^ ANC prevents maternal, fetal morbidity and mortality by providing information regarding danger signs, childbirth preparation, early detection and timely intervention of pregnancy related health issues and prevent complications.^3^

The redirection of resources from maternal health services towards COVID-19 containment has contributed to increased risk of maternal morbidity and mortality.^4^ Despite this, the impact of COVID-19 on pregnant women is less explored especially in a country like Nepal.

This study aimed to find the prevalence of irregular ANC attendance among pregnant women during COVID-19 pandemic in a tertiary care centre.

## METHODS

A descriptive cross-sectional study was conducted among pregnant women attending the Department of Obstetrics and Gynaecology of the tertiary care centre between 23 July 2021 and 5 September 2021. Ethical approval was granted by the Institutional Review Committee of KIST Medical College (Reference number: 077/078/67). All the pregnant women visiting the tertiary care center for their routine ANC check-ups were included in the study. The patients not willing to participate and give their consent, patients who came for their first ANC visits without any prior ANC visit history, and those with chronic debilities were excluded from the study. A convenience sampling method was used for the study. The sample size was calculated using the following formula:


$$\begin{array}{ll} \text{n} & ={\text{Z}}^{2}\times \frac{\text{p}\times \text{q}}{{\text{e}}^{\text{2}}}\\ & =1.96^{2}\times \frac{0.5\times 0.5}{0.07^{2}}\\ & =196 \end{array}$$

Where,

n= minimum required sample size
Z= 1.96 at 95% Confidence Interval (CI)
p= prevalence taken as 50% for maximum sample size calculation
q= 1-p
e= margin of error, 7%

Hence, a sample size of 196 was taken in the study. The data was collected through face-to-face interviews with the participants and were filled in the questionnaire. The semi-structured questionnaire was developed by the research team after relevant literature reviews and expert opinion. Pretesting of the questionnaire was done.

The questionnaire included socio-demographic and obstetric characteristics, reasons for missing an ANC visit, COVID-19 safety precautions followed during the visit, changes in medical professional behaviour, and impact of COVID-19 in ANC visits. Nepal follows the WHO standard of initiating ANC within the first 4 months of pregnancy and at least 4 ANC visits in an uncomplicated pregnancy.^5^ Therefore, at least four ANC visits was regarded as the standard for reference in our study.

Data entry and analysis were done by using IBM SPSS Statistics 21.0. Point estimate and 95% CI were calculated.

## RESULTS

Among 196 pregnant women who visited the tertiary care center for ANC, 49 (25%) (18.96-31.06, 95% CI) had irregular ANC attendance. A total of 28 (57.14%) were attending four or more ANC visits, while 15 (30.61%) were attending their third ANC visits and six (12.24%) their second ANC visits.

Contracting COVID-19 during hospital visits was the major concern of 38 (77.55%) participants who missed their ANC visits. Nine (16.33%) thought that suboptimal services might be provided by the health professions, four (8.16%) had cancellation of the scheduled ANC visit and one (2.04%) had unavailability of medicines and laboratory facilities.

Among the patients with irregular ANC visits, lack of transportation service was seen in 31 (63.26%), fear of contracting COVID-19 in 20 (40.82%) amd two (4.08%) preferred home isolation. It was found that four (8.16%) used telehealth service for their pregnancy-related concerns. The majority of the women, 31 (63.26%) were from the age group of 2534 years. Among them, 15 (30.61%) had secondary level education or higher while one (2.04%) participant had not received any formal education. The majority 34 (69.39%) were multigravida (Table 1).

**Table 1:** Socio-demographic profile and parity of pregnant women who missed ANC visit (n= 49).

Variables	Category	n (%)
Age	15-24	14 (28.57)
	25-34	31 (63.27)
	>35	4 (8.16)
Residence	Village	8 (16.33)
	Urban	41 (83.67)
Religion	Hindu	34 (69.39)
	Buddhist	8 (16.33)
	Christian	5 (10.20)
	Muslim	1 (2.04)
	Others	1 (2.04)
Education level	Nonformal	1 (2.04)
	Primary	9 (18.36)
	Secondary	21 (42.86)
	Higher secondary	15 (30.61)
	Undergraduate and more	3 (6.12)
Occupation	Housewife	35 (71.43)
	Self-employed	9 (18.36)
	Farming	1 (2.04)
	Government job	1 (2.04)
	Others	3 (6.12)
Husband's occupation	Farming	3 (6.12)
	Self-employed	4 (8.16)
	Labourer	7 (14.29)
	Foreign worker	5 (10.20)
	unemployed	2 (4.08)
	Others	28 (57.14)
Type of family	Nuclear	33 (67.35)
	Joint	16 (32.65)
Gravida	Primigravida	15 (30.61)
	Multigravida	34 (69.39)

A total of 45 (91.83%) had taken the recommended medications and vaccination during the pregnancy and 44 (89.79%) received their supplementation of folic acid. Similarly, iron was taken by 45 (91.83%), calcium by 44 (89.79%), both doses of tetanus toxoid injection by 46 (93.87%), and deworming tablet by 30 (61.22%) participants.

All ie 49 (100%) followed precautions and safety measures to prevent themselves from contracting COVID-19 during their ANC visits. The use of masks and handwashing was practiced by 49 (100%) women. Use of sanitizers was practiced by 47 (95.91%). Safe social distancing was practiced by 41 (83.67%) and a face shield was used by 23 (46.93%) women.

## DISCUSSION

Our study reported that 25% of the pregnant women missed their regular ANC visit among which 57.14% were attending four or more ANC visits. COVID-19 poses an equal risk to people irrespective of their age and gender but pregnant females are amongst the most vulnerable population due to the physiological changes in the immune and cardiovascular systems. Hence, they are also more prone to develop serious symptoms and complications after being infected with this respiratory virus. The pandemic has resulted in pregnant women facing difficulties in accessing maternal health care that including restrictions, transportation problems, and anxiety about contracting COVID-19 and possibly transmitting the virus to their unborn babies. The travel restrictions have further made it difficult to reach to the health institutions and those who had visited the health centres complained of not receiving proper treatment.^6^

This study was carried out to find out how much COVID-19 affected on the utilization of ANC services among pregnant women in a tertiary care hospital. Out of 196 participants in the study, the result showed that 49 (25%) of pregnant women missed their ANC visits. In a study done in Northeast Ethiopia, it was found that (55.5%) of respondents missed or were late to start antenatal services during the COVID-19 pandemic. The majority (56.48%), attributed this to fear of contracting COVID-19, followed by disruption and diversion of maternal services to COVID-19 72 (33.33%).^2^

Compared to Northeast Ethiopia, our study showed higher ANC coverage with only 49 (25%) missing their ANC visits. This may be attributed to the age of the respondents and their educational status, sociodemographic characteristics, parity, occupation, ethnicity, their knowledge, understanding, and awareness about the importance of ANC service utilization during pregnancy, prior experience about the benefits of ANC, urban residency, financial conditions, family support and also due to their awareness of safe practices to prevent contracting COVID-19 during their ANC Visits and accessibility to hospitals.

In a study done in Nepal to assess the impact of ANC visits on maternal and perinatal outcome before the pandemic, it was found that only (6.5%) didn't attend their ANC visits.^7^ This huge contrast in findings when compared with the result of our study where 49 (25%) pregnant female missed their ANC visits shows the problem of COVID-19 on ANC visits.

We studied the age, parity, education level and occupation level of the pregnant women and ANC attendance. The pregnant women during their ANC visits have taken their recommended doses of iron supplementation, calcium, deworming tablets, folic acid, vitamin B complex and immunization with tetanus toxoid. Similarly, respondent's perception on health services provided by the doctors in their ANC visits after pandemic was also found to be changed where the participants mentioned of less time for consultation, minimal physical examination, less attention and leaving the consultation on midway to attend other patients and phone calls.

The findings of the study showed that all of the pregnant women during their ANC visits have followed various safety measures to prevent contracting COVID-19. This finding is higher than those reported from the studies done in North West Ethiopia and Nigeria reporting only (47.6%) and (30.3%) respectively.^8,9^ This might be due to their good knowledge, understanding and awareness of safe practices about prevention measures from contracting COVID-19. Compulsory use of mask, sanitizer, face-shield, proper handwashing and increased price in transportation has made the situation difficult for the pregnant women and their family members since these resources are scarce and overpriced.

In our study, it was found that only 4 (8.16%) participants who missed their ANC visits used telehealth services regarding ANC. However, COVID-19 has highlighted the importance of such online telemedicine. Telephone triage and online telehealth consultation with the health professionals can prevent the frequent visits by the pregnant women to the hospital for a minor condition which could have been easily solved with proper history taking. This not only limits the exposure of the pregnant women to COVID-19 during the hospital visit but they can also have access to the health professionals almost in any situations. It could also be used to increase awareness regarding importance of ANC visits, preventive measures against COVID-19 and quarantine. Telemedicine could give an edge to the ANC service utilization by pregnant women.^10^

Regardless of the difficult circumstances created due to COVID-19, our result found that 45 (91.83%) who missed their ANC visits considered ANC visits were important even in pandemic and among them 44 (89.79%) participants had their family support to attend ANC visits.

The limitation of the study was small sample size, single centre study and the study population excluded women with chronic disease and debilities as a result of which complete representation could not be done in the study.

## CONCLUSIONS

The prevalence of irregular antenatal care attendance during the COVID-19 pandemic was lower than other studies done in similar settings. The pandemic has caused difficulties for pregnant women to access maternal health care like ANC. Our study could help in the development and implementation of new policies to ensure regular ANC visits. Increasing awareness about the importance of recommended ANC visits to pregnant women, preventive and safety measures to avoid contracting COVID-19 during their hospital visits can be practised to encourage and ensure regular ANC.
